# Probing consequences of anion-dictated electrochemistry on the electrode/monolayer/electrolyte interfacial properties

**DOI:** 10.1038/s41467-020-18030-6

**Published:** 2020-08-21

**Authors:** Raymond A. Wong, Yasuyuki Yokota, Mitsuru Wakisaka, Junji Inukai, Yousoo Kim

**Affiliations:** 1 Surface and Interface Science Laboratory, RIKEN, 2-1 Hirosawa, Wako, Saitama, 351-0198 Japan; 2 PRESTO, Japan Science and Technology Agency (JST), 4-1-8 Honcho, Kawaguchi, Saitama, 332-0012 Japan; 3 Graduate School of Engineering, Toyama Prefectural University, 5180 Kurokawa, Imizu, Toyama, 939-0398 Japan; 4 Clean Energy Research Center, University of Yamanashi, 4-3-11 Takeda, Kofu, Yamanashi, 400-8510 Japan

**Keywords:** Electrochemistry, Surface spectroscopy

## Abstract

Altering electrochemical interfaces by using electrolyte effects or so-called “electrolyte engineering” provides a versatile means to modulate the electrochemical response. However, the long-standing challenge is going “beyond cyclic voltammetry” where electrolyte effects are interrogated from the standpoint of the interfacial properties of the electrode/electrolyte interface. Here, we employ ferrocene-terminated self-assembled monolayers as a molecular probe and investigate how the anion-dictated electrochemical responses are translated in terms of the electronic and structural properties of the electrode/monolayer/electrolyte interface. We utilise a photoelectron-based spectroelectrochemical approach that is capable of capturing “snapshots” into (1) anion dependencies of the ferrocene/ferrocenium (Fc/Fc^+^) redox process including ion-pairing with counter anions (Fc^+^–anion) caused by differences in Fc^+^–anion interactions and steric constraints, and (2) interfacial energetics concerning the electrostatic potential across the electrode/monolayer/electrolyte interface. Our work can be extended to provide electrolyte-related structure-property relationships in redox-active polymers and functionalised electrodes for pseudocapacitive energy storage.

## Abstract (web summary)

Conveying electrochemistry in terms of the electrode/electrolyte interfacial properties remains challenging. Here, the authors employ a surface-bound molecular probe and photoelectron spectroscopy to peer into the anion-dictated and potential-induced interfacial electronic and structural properties.

## Introduction

Central to electrochemistry is the constant pursuit to elucidate the underlying factors that govern the electrochemical response. The motivation stems from the desire to augment the functionality of devices by controllably altering key parameters such as the redox potential, reaction pathway, reversibility and stability. However, achieving this requires an intimate knowledge of the interfacial properties as this affects the charge transfer process and thus, the overall electrochemical response. This fuels the desire to obtain structure–property relationships that sufficiently encompasses the intricacies of the electrochemical environment at the electrode/electrolyte interface^1–4^.

**Fig. 1:**
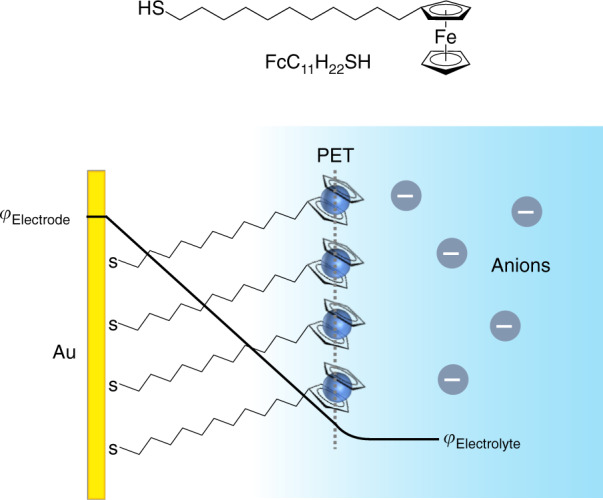
Model system used as a molecular probe. Molecular structure of 11-ferrocenyl-1-undecanethiol (top panel) that form ferrocene-terminated alkanethiol self-assembled monolayers (Fc SAM) on Au. Schematic representation (bottom panel) of the Fc SAM electrode/monolayer/electrolyte interfacial region showing the interfacial potential distribution (*φ*) from Au metal electrode to electrolyte. Plane of electron transfer is denoted as PET.

To this end, surface-confined redox molecules such as ferrocene-terminated alkanethiol self-assembled monolayers (Fc SAM) on Au (Fig. 1) constitute an attractive molecular probe for correlative studies into interfacial electrochemistry and the physicochemical properties of the interface^4–6^. With relevance to bio(chemical) sensing^7^, redox-induced micro-actuators^8^ and molecular electronics^9^, the corresponding Fc termini can switch between neutral Fc (ferrocene) and cationic Fc^+^ (ferrocenium) redox states via single-electron transfer and ion-pairing reactions with anions: (Fc)_SAM_ + X^−^_aq_ ⇆ (Fc^+^–X^−^)_SAM_ + e^−^^10,11^. Therefore, the anion should play an important role in the Fc/Fc^+^ redox process and has been shown to influence the apparent formal potential (*E*^*o*^′) and reversibility owing to the influence of anion solvation on the behaviour of ion pairing^12,13^. Concomitantly, this implies that the anion should affect the microscopic ion-pair structures^14^ and the related structural changes including monolayer thickness^15,16^ and molecular orientation^17^. While there are techniques that can probe such structural changes, we will show for the first time, the ability to also discriminate the anion-dictated Fc SAM interfacial electronic properties.

A related aspect is the interfacial energetics as first envisioned by Helmholtz and later expanded by Gouy–Chapman and Stern^4^. The electrified electrode/electrolyte interface consists of a region with a linear potential gradient (inner Helmholtz plane) followed by the outer Helmholtz plane (diffuse layer) where the potential profile decays monotonically into the electrolyte. In the presence of a SAM, the linear potential portion is dependent on the length of the molecules adsorbed on the electrode surface (Fig. 1)^18–20^. Existing studies aimed at corroborating these theories have predominantly relied on systematically varying the SAM structure. For instance, by varying the length of *n*-alkanethiols and measuring cyclic voltammetry (CV), Porter et al. showed that the role of interfacial capacitance can be indirectly inferred^21^. Eggers et al. demonstrated the relationship between the apparent formal potential and the height of the Fc termini relative to a co-adsorbed non-electroactive SAM^22^. While varying the SAM structure can be an effective approach, methods that can directly interrogate the influence of the interfacial potential distribution have thus far, been very limited.

**Fig. 2:**
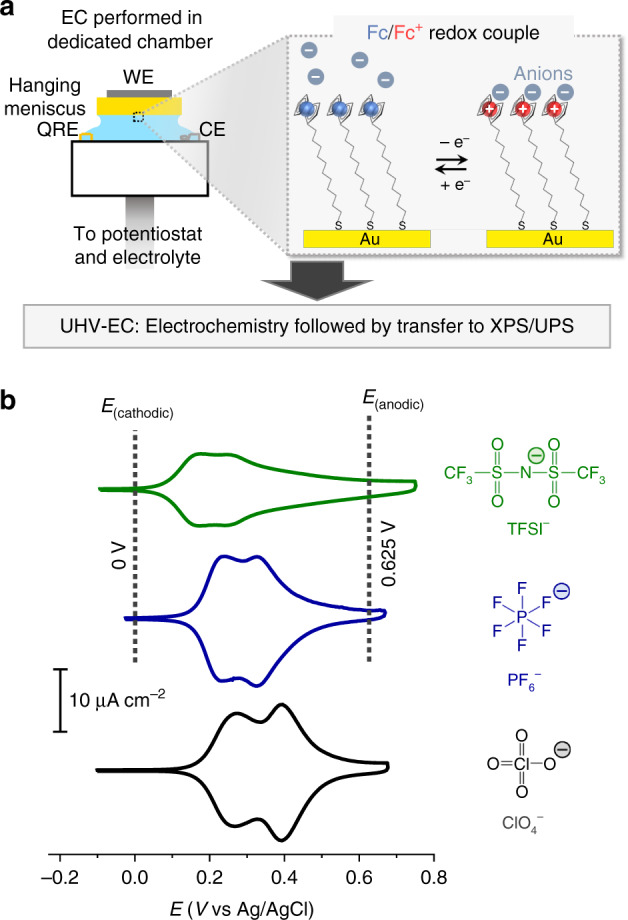
Electrochemical cell setup and anion-dictated electrochemical responses. **a** A schematic of the electrochemical (EC) cell located inside a dedicated chamber where electrochemical measurements are performed under Ar atmosphere followed by evacuation and transfer to XPS/UPS chamber for analysis. The WE, QRE and CE denote the working, quasi reference and counter electrodes, respectively. See Methods and Supplementary Fig. 1 for details. **b** Steady state cyclic voltammograms (CVs) of Fc SAM on Au(111) performed in 0.1 M NaTFSI, NaPF_6_ and NaClO_4_ at a scan rate of 50 mV s^−1^. The vertical dotted lines indicate the potentials 0.625 and 0 V, denoted as *E*_(anodic)_ and *E*_(cathodic)_, respectively which are the potentials the electrode was polarised for the EC-XPS/UPS measurements.

Our work aims to address these challenges by employing photoelectron spectroscopy in the X-ray and ultraviolet wavelengths (XPS/UPS). Based on the photoelectric effect, XPS/UPS detects the kinetic energy of ejected photoelectrons to reveal the electronic and chemical environment of atoms at the sample surface. However, an intrinsic obstacle is the need for vacuum conditions in order to obtain useful detection probabilities^23^. A workaround is to employ a so-called pseudo in situ UHV-EC approach originally conceived by Hubbard and coworkers^24–29^ to link the electrochemical (solution) and XPS/UPS (ultrahigh vacuum, UHV) environments (see Methods). We note that alternatively, true in situ XPS can offer another avenue to explore solid/liquid interfaces^1,30–36^, while each approach has noteworthy trade-offs such as signal-to-noise versus temporal resolution (see Supplementary Note 1). The UHV-EC approach used in the present study involves electrode immersion (Fig. 2a and Supplementary Fig. 1) where electrochemical measurements are performed in a dedicated chamber under Ar atmosphere. This is followed by removal under potential control and then transfer to vacuum to enable a “snapshot-like” analysis into the electrochemical-induced changes. Notable work using UHV-EC by Kolb and Hansen attributed the observation of systematic XPS binding energy shifts with electrode potential as an indicator that the electrochemically-induced changes (i.e. double layer) can be conserved following electrode immersion^1,2,26,37,38^.

Herein, we interrogate the Fc SAM molecular probe and show how the anion-dictated electrochemistry is conveyed in terms of the electronic and structural properties of the electrode/monolayer/electrolyte interface. We predominantly focus on two anions, namely a bulky organic anion ([CF_3_SO_2_]_2_N^−^, denoted as TFSI^−^) and an inorganic anion (PF_6_^−^), which should invariably affect the ion–ion and ion–solvent interactions owing to differences in ionic size, charge density, polarisability and flexibility^39^. We use a spectroelectrochemical approach consisting of XPS/UPS combined with an electrochemical cell (denoted hereon as EC-XPS/UPS) to electrochemically control the Fc SAM followed by spectroscopic observations into the anion-dictated aspects of the Fc/Fc^+^ redox process including Fc^+^–X^−^ ion-pairing, monolayer thickness, Fe oxidation state and valence structure. A key aspect of our findings is related to the TFSI^−^ anion which uniquely restricts the Fc^+^ reaction yield, thereby creating the conditions where the influence of the electrostatic potential across the electrode/monolayer/electrolyte interface is readily observable via concomitant core and valence-level binding energy shifts. The work presented here underscores how “beyond cyclic voltammetry” approaches consisting of vacuum-based methods can be effective in ascertaining the intricacies of the electrode/electrolyte interface.

## Results

### Anion-dictated electrochemical responses

Cyclic voltammograms (CVs) of Fc SAM on Au(111) in 0.1 M NaTFSI, NaPF_6_ and NaClO_4_ (Fig. 2b) show that in addition to the apparent formal potentials (*E*^*o*^′), the nature of the anion also influences the degree of conversion to Fc^+^ (reaction yield). Namely, the apparent formal potentials (Table 1) indicates that oxidation initiates at lower anodic potentials with TFSI^−^ (0.180 V) followed by PF_6_^−^ (0.240 V) and ClO_4_^−^ (0.270 V), respectively. The integral charge from the CVs indicates the degree of conversion to Fc^+^ (*Γ*_Fc→Fc_^+^, Table 1 and Supplementary Fig. 2). The *Γ*_Fc→Fc_^+^ exhibited by TFSI^−^ is lower (3.1 × 10^−10^ mol cm^−2^) in comparison to PF_6_^−^ and ClO_4_^−^, which are similar (4.6–4.7 × 10^−10^ mol cm^−2^), while it should be noted that the theoretical Fc SAM coverage is 4.5 × 10^−10^ mol cm^−2^ (assuming close-packed Fc with a diameter of 0.66 nm)^5,40^. All of the CVs are reversible (negligible anodic-cathodic peak separations) and all of the peak currents increase linearly with scan rate (Supplementary Fig. 3) thus, confirms that the Fc SAM are surface-confined to the Au electrode. It should be noted that all of the CVs exhibit non-ideal behaviour as seen by the multiple CV peaks that are commonly observed in close-packed Fc SAM^41,42^, and can be attributed to reasons including local heterogeneity and intermolecular interactions (see Supplementary Note 2 on non-ideal behaviour).

**Table 1:** Summary of Fc SAM electrochemical CV data for TFSI^−^, PF_6_^−^ and ClO_4_^−^ from Fig. 2b.

Anion	Apparent formal potential (*E*^*o*^′) (V vs. Ag/AgCl)^a^	Integral charge transferred (*Γ*_Fc→Fc_^+^) (mol cm^−2^)^b^	*Γ*_Fc→Fc_^+^/*Γ*_Thereotical_ (ratio)^c^
TFSI^−^	0.180 (0.240)	3.1 × 10^−10^	~0.70
PF_6_^−^	0.240 (0.330)	4.6 × 10^−10^	~1.0
ClO_4_^−^	0.270 (0.400)	4.7 × 10^−10^	~1.0

^a^*E*^*o*^′ is the mean of the first set of anodic and cathodic peaks, while the parenthesis corresponds to the second set of peaks.

^b^Used linear background subtraction between 0 and 0.625 V.

^c^*Γ*_Theoretical_ is 4.5 × 10^−10^ mol cm^−2^ (assuming close-packed Fc with a diameter of 0.66 nm)^40^.

### Occurrence of Fc^+^–X^−^ ion-pairing and its reversibility

Subsequent spectroelectrochemical characterisations of the Fc SAM were performed on the pristine sample and after polarisation at 0.625 and 0 V (denoted as *E*_(anodic)_ and *E*_(cathodic)_ in Fig. 2b, respectively). These potentials provide an equal footing of comparison since *E*_(anodic)_ and *E*_(cathodic)_ can be expected to predominantly yield (neutral) Fc and oxidised (cationic) Fc^+^ redox states, respectively. We primarily focus on contrasting TFSI^−^ and PF_6_^−^ due to their distinct differences, whereas ClO_4_^−^ exhibits comparable CV characteristics to PF_6_^−^^40^.

**Fig. 3:**
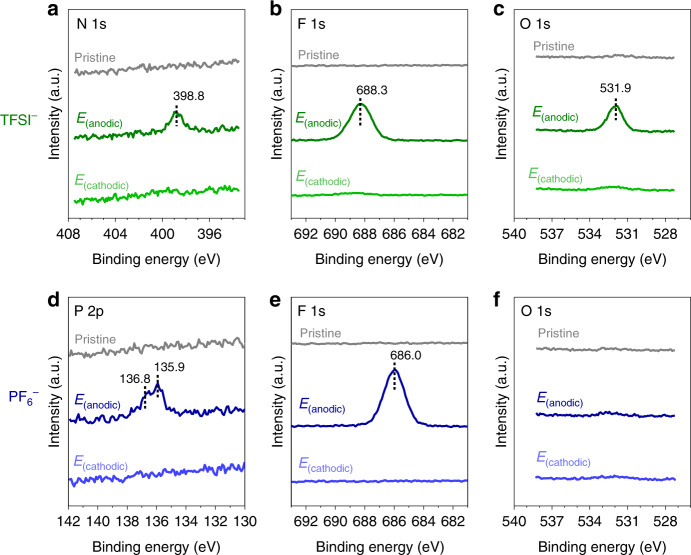
Occurrence of Fc^+^–X^−^ ion-pair formation and its reversibility. EC-XPS showing the anion spectra for pristine Fc SAM and following polarisation at 0.625 and 0 V, denoted as *E*_(anodic)_ and *E*_(cathodic)_ respectively, corresponding to 0.1 M NaTFSI **a** N 1s, **b** F 1s, **c** O 1s and 0.1 M NaPF_6_, **d** P 2p, **e** F 1s and **f** O 1s. Note that the S 2p spectra is shown in Fig. 7a, b. XPS performed utilising Al *Kα*, *hv* = 1486.6 eV at room temperature.

The Fc/Fc^+^ redox reaction can be confirmed by observing the reversibility of Fc^+^–X^−^ ion-pair formation and the structural changes of the electrode/monolayer/electrolyte interface. Oxidation to Fc^+^ is accompanied by the formation of Fc^+^–X^−^ ion-pairs due to the charge compensation of the charged Fc^+^ by counter anions in the electrolyte^10,12,14^. After *E*_(anodic)_, XPS shows the emergence of anion-related features resembling TFSI^−^ (Fig. 3a–c, N 1s, F 1s, O 1s) and PF_6_^−^ (Fig. 3d, e, P 2p and F 1s). These features diminish following *E*_(cathodic)_ corresponding to the conversion back to Fc, thus indicates the reversibility of Fc^+^–X^−^ ion pair formation. For both TFSI^−^ and PF_6_^−^, the XPS-determined stoichiometry (Supplementary Table 1) indicates the formation of 1:1 Fc^+^–X^−^ ion pairs. However, in the case of TFSI^−^, it is important to note that the Fe: N: F atomic ratio of 1.2: 1.0: 6.0 is in line with *Γ*_Fc→Fc_^+^ obtained from the CV results (Fig. 2b) meaning that ~70–80% of the Fc can be oxidised to Fc^+^. We note that this is consistent with the deconvoluted Fe 2p spectra (Supplementary Fig. 4 and vide infra). The partial conversion to Fc^+^ suggests steric constraints of the bulkier TFSI^−^ in comparison to PF_6_^−^ (effective ionic radius of 0.33 and 0.25 nm, respectively)^43–45^. Complementary experiments examining competitive ion-pairing (mixed anions in Supplementary Fig. 5) and Fc SAM diluted with non-electroactive *n*-alkanethiols (Supplementary Fig. 6) supports this reasoning. Moreover, *E*_(anodic)_ is met by a larger increase in effective monolayer thickness (Supplementary Fig. 7 and Supplementary Note 3 via Au 4f attenuation) with TFSI^−^ (22.0 ± 0.7 Å) than PF_6_^−^ (19.9 ± 0.5 Å), which implies differences in Fc^+^–X^−^ ion-pair structure/size.

Our spectroelectrochemical observations are in line with electrochemical quartz crystal microbalance (EQCM) studies that have suggested that reversible 1:1 ion-pairing occurs in the presence of ClO_4_^−^ anions^46,47^. Therefore, similar to the case with ClO_4_^−^, the formation of 1:1 Fc^+^–X^−^ contact ion-pairs is attributed to the hydrophobic (weakly solvating) nature of TFSI^−^ and PF_6_^−^, which strongly favours ion-pair formation to stabilise the charged Fc^+^. In addition, the O 1s spectra for PF_6_^−^ (contains no O atoms) is largely featureless following *E*_(anodic)_ and *E*_(cathodic)_, while in the case of TFSI^−^, O 1s features are prominent only after *E*_(anodic)_ which is attributed to TFSI^−^ forming the abovementioned Fc^+^–X^−^ ion pairs. These O 1s spectral observations suggest that the immersion process “unzips” and eliminates weakly-interacting, excess electrolyte and that water is not strongly interacting with the immersed Fc SAM electrode^48^. We further note that (Na^+^) cation involvement in terms of charge compensation is found to be negligible at the potentials analysed (Supplementary Fig. 8, Na 1s and Na 2p spectra). In all, the observation of reversible 1:1 Fc^+^–X^−^ ion-pairing indicates that the electrochemically-induced changes are conserved following electrode immersion and transfer.

**Fig. 4:**
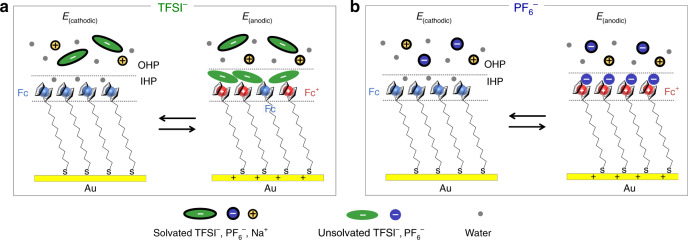
Schematic representation of electrode/monolayer/electrolyte interface upon *E*_(anodic)_ and *E*_(cathodic)_. **a** TFSI^−^ and **b** PF_6_^−^. Solvated ions are denoted by the encased black outline. IHP and OHP denote the inner and outer Helmholtz planes, respectively.

In light of the abovementioned observations, we can summarize the structural aspects of the electrode/monolayer/electrolyte interface as schematically shown in Fig. 4. Polarisation at *E*_(anodic)_ yields significant changes where the charged Fc^+^ are effectively screened by counter anions at the inner Helmholtz plane resulting in a robust adlayer of 1:1 Fc^+^–X^−^ contact ion-pairs, and concomitantly leads to the increase in monolayer thickness. In terms of anion dependencies, in the case of PF_6_^−^, there are exclusively 1:1 Fc^+^–PF_6_^−^ ion pairs, whereas in the case of TFSI^−^, 1:1 Fc^+^–TFSI^−^ ion pairs coexist with neutral Fc due to steric constraints^45^. On the other hand, for *E*_(cathodic)_, the inner Helmholtz planes are structurally similar for both TFSI^−^ and PF_6_^−^ and consists of weakly-interacting water, while any ions that are present are expected to be solvated owing to the negligible surface charge.

However, despite reversible ion pairing, the anion-dictated apparent formal potentials are a reflection of differences in the ion–solvent and ion–ion interaction strengths. The resulting competition between the two dictates the favourability or tendency of Fc^+^–X^−^ ion pairing. The tendency of ion-pairing can be empirically expressed as *K* (ion-pair formation constant) in the following Nernst relation^10^:

1 $$E \,=\, E^o \,+\, 2.303\frac{{\rm{RT}}}{F}{\rm{log}}\frac{{[{\rm{Fc}}^ + - X^ - ]}}{{\left[ {\rm{Fc}} \right]K}} \,-\, 2.303\frac{{\rm{RT}}}{F}{\rm{log}} [X^ - ],$$

where *E*^*o*^ is the standard reduction potential and all other parameters have their usual meanings. Therefore, the apparent formal potential should depend on both *K* and anion concentration [X^−^]. A plot of the apparent formal potential as a function of anion concentration (Supplementary Fig. 9) shows that all anions exhibit similar slopes of 2.303RT/*F* (equivalent to ~60 mV per decade), meaning that the difference in the vertical intercepts originates from the *K* parameter. Based on the difference of the vertical intercepts (~60 mV), we can ascertain that the *K* for TFSI^−^ is 10 times larger than in the case of PF_6_^−^. We note that alternatively, the ion-pairing tendencies and apparent formal potentials can be conceptually rationalised by invoking Pearson’s hard-soft acid-base theory to explain Fc^+^–X^−^ interactions based on the differences in the Lewis basicity of the anion^49^.

### Electronic aspects of electrode/monolayer/electrolyte interface

**Fig. 5:**
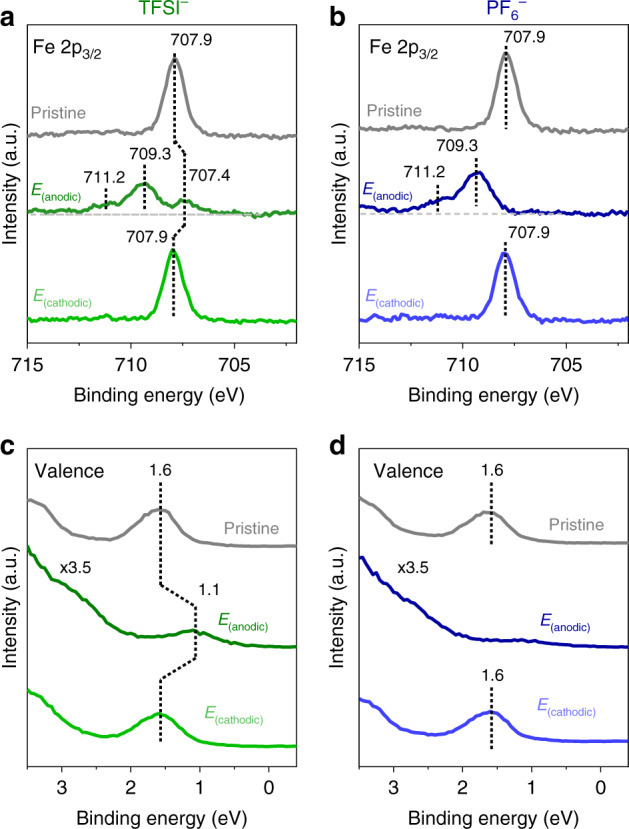
Fc/Fc^+^ redox centre and electrochemically-induced binding energy shifts. EC-XPS/UPS of pristine Fc SAM and following polarisation at *E*_(anodic)_ and *E*_(cathodic)_. **a**, **b** XPS Fe 2p_3/2_ region for TFSI^−^ (left panel) and PF_6_^−^ (right panel). The deconvoluted Fe 2p_3/2_ spectra for *E*_(anodic)_ is shown in Supplementary Fig. 4. **c**, **d** UPS valence spectra for TFSI^−^ (left panel) and PF_6_^−^ (right panel), respectively. The horizontal dashed lines act as a guide to the eyes.

We now move to the electronic aspects of the electrode/monolayer/electrolyte interface. The XPS Fe 2p and UPS valence spectra (Fig. 5 and Supplementary Fig. 10) confirms that the pristine Fc SAM are indeed un-oxidised (neutral) Fc^50^. The sharp symmetry of the Fe 2p_3/2_ peak at 707.9 eV (Fig. 5a, b) reflects the (low spin) diamagnetic character of Fe(II)^50,51^. Meanwhile, the UPS spectra shows a peak at 1.6 eV (Fig. 5c, d and Supplementary Fig. 11) ascribed to the highest occupied molecular orbital (HOMO) of Fc, comprised of the six 3d electrons from Fe^51,52^. Based on the geometry of the cyclopentadienyl (Cp) ligands, Fc has the electronic configuration (a_1_′)^2^(e_2_′)^4^ and (a_1g_)^2^(e_2g_)^4^ for the eclipsed (D_5h_) and staggered (D_5d_) conformations, respectively^53,54^. The XPS Fe 2p (core level) and UPS valence spectra can be tracked together because the Fc HOMO is localised to the Fe heteroatom causing them to be strongly correlated^55,56^.

Subsequent polarisation at *E*_(anodic)_ leads to significant spectral changes with anion dependencies (Fig. 5). In both TFSI^−^ and PF_6_^−^, oxidation is evidenced by the Fe 2p_3/2_ features at higher binding energy (~709.3 eV in Fig. 5a, b and Supplementary Fig. 4) and is consistent with an increase in the Fe oxidation state to Fe(III). Also consistent with Fe(III) is the broadening and asymmetry of the Fe 2p_3/2_ peaks (notable shoulder at 711.2 eV) owing to final state effects including multiplet splitting caused by the presence of an unpaired valence electron in the (low spin) paramagnetic Fe(III)^51,55^. Note that in TFSI^−^, the remaining Fe(II) features (707.4 eV) indicate residual Fc. Meanwhile, oxidation is also evident in the valence spectra (Fig. 5c, d), where the Fc HOMO peak diminishes in TFSI^−^ (indicates residual Fc) and is clearly no longer prominent in the case of PF_6_^−^. The HOMO of the oxidised Fc^+^ should be located at higher binding energy due to factors including increased Coulombic attraction but is likely overlapped by the anion and SAM-related features (i.e. alkyl chain and Au substrate) at binding energies >2 eV (Supplementary Fig. 11)^57^. The extended valence spectra further supports differences in the interfacial ion-pair structures as seen by the pronounced TFSI^−^ spectral features in the binding energy range 4–12 eV (Supplementary Fig. 11b), whereas in PF_6_^−^, the SAM features clearly remain. These observations show the uniqueness of the Fc^+^–TFSI^−^ ion-pair structure which can strongly screen the SAM/Au spectral features.

In the case of TFSI^−^, a key observation is that the Fe(II) features in the Fe 2p core level and Fc HOMO (valence spectra) show systematic binding energy shifts with respect to the polarisation potential (*E*_(cathodic)_ and *E*_(anodic)_). Specifically, the Fe(II) peak in the Fe 2p_3/2_ spectra (Fig. 5a) is centred at 707.9 and 707.4 eV for *E*_(cathodic)_ and *E*_(anodic)_, respectively. Similarly, in the valence spectra (Fig. 5c), the Fc HOMO peak is centred at 1.1 and 1.6 eV for *E*_(cathodic)_ and *E*_(anodic)_, respectively. Taken together, this corresponds to a ~0.8 eV/V relationship between binding energy shift and electrode potential. It should be noted that in terms of the intermediate potentials, EC-XPS/UPS measurements performed at increasing potentials with ClO_4_^−^ (Supplementary Fig. 12) corroborates that the observed systematic binding energy shifts are linear.

These systematic XPS/UPS binding energy shifts are attributed to the influence of the electrostatic potential across the electrode/monolayer/electrolyte interface^2,20^. The electrostatic potential profile is expected to be linear in accordance with the parallel plate capacitor model (Fig. 1)^19,22^ where the majority of the potential drop occurs within the monolayer (Supplementary Note 4 and Supplementary Fig. 13). Specifically, the binding energy shifts reflect the change in the potential difference between *φ*_Electrode_ and *φ*_Electrolyte_ (Fig. 1), and is related to the electrode potential (*ΔE*) as shown below:^19,58^

2 $${\Delta}E \,=\, {\Delta}(\varphi _{\rm{Electrode}} \,-\, \varphi _{\rm{Electrolyte}}).$$

**Fig. 6:**
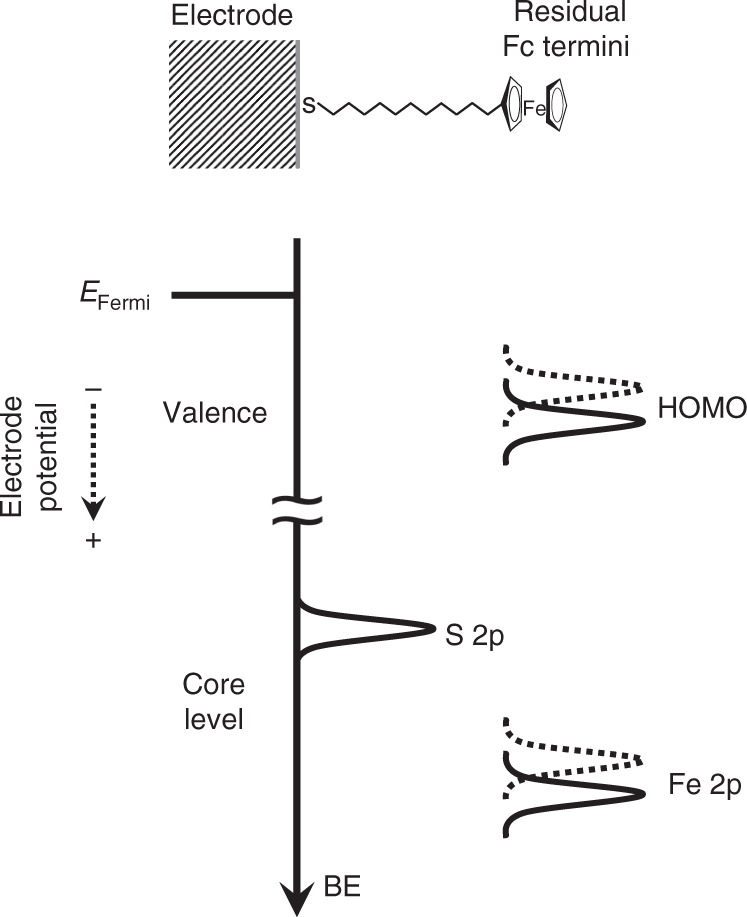
Electrochemically-induced core and valence-level binding energy shifts. A schematic showing the relationship between Fermi level (*E*_Fermi_), electrode potential and the XPS/UPS binding energy shifts. BE denotes binding energy. The dotted lines denote the direction of the core and valence-level binding energy shifts that occur upon positive polarisation.

**Fig. 7:**
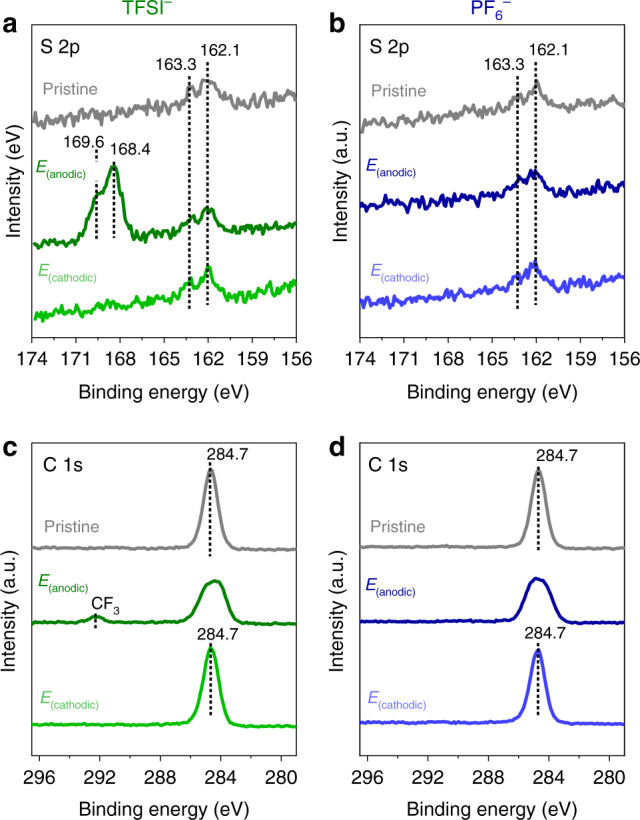
Positional influence across electrode/monolayer/electrolyte interface. EC-XPS of the pristine Fc SAM and following polarisation at *E*_(anodic)_ and *E*_(cathodic)_ corresponding to the S 2p spectra for **a** TFSI^−^ and **b** PF_6_^−^. C 1s spectra for **c** TFSI^−^ and **d** PF_6_^−^. Note in the case of TFSI^−^ following *E*_(anodic)_, the features in the S 2p (169.6 and 168.4 eV) and C 1s (292.3 eV) spectra are due to the TFSI^−^ anion forming Fc^+^–TFSI^−^ ion pairs (see text)^51^.

The binding energy shifts are observed because the binding energy is measured with respect to the Fermi level (*E*_Fermi_) of the Au substrate which is the same entity as the electrode potential (illustrated in Fig. 6). Meanwhile, on the other side of the interface, the Fc termini are electronically decoupled from the substrate, and therefore is referenced to the electrolyte (*φ*_Electrolyte_)^2,20^. The net effect of positive potential yields a lower binding energy, while negative potential yields higher binding energy (Fig. 6)^20^. Taken together, this means that the position within the monolayer relative to the Au electrode affects the binding energy shift^20^. This is consistent with the negligible binding energy shift in the S 2p spectral contributions from the bound thiolate (doublet at 163.3 and 162.1 eV in Fig. 7a, b)^50^, irrespective of the polarisation potential because the anchoring S atoms are in close proximity to the Au electrode. It should be noted that following *E*_(anodic)_ in TFSI^−^, there is an additional S 2p doublet at 169.6 and 168.4 eV (Fig. 7a)^51^ ascribed to the presence of TFSI^−^ anions forming ion-pairs (vide supra). The positioning within the monolayer is also ascribed as a contributing factor that causes the broadening of the C 1s spectra upon *E*_(anodic)_ attributed to the C atoms located along the alkyl chain (Fig. 7c, d). We note that the ~0.8 eV/V relationship is reasonably close to the expected 1 eV/V relationship^2,26,38^, and deviations can be caused by non-idealities such as buried Fc that have been reported to exist within the monolayer^42,59^.

## Discussion

It is important to reiterate the role of TFSI^−^ in creating an environment that inhibits the full conversion to Fc^+^ leading to conditions where at XPS/UPS can readily probe the influence of the electrostatic potential across the electrode/monolayer/electrolyte interface. In addition, the Fe heteroatom is positioned at the edge of the electrode/monolayer/electrolyte interface which yields a clear electrostatic potential difference that can be readily probed. On the other hand, the interfacial environment in the case of PF_6_^−^, causes the complete Fc/Fc^+^ conversion at the potentials analysed (*E*_(cathodic)_ and *E*_(anodic)_) resulting in a discrete binding energy shift associated with oxidation, and thus masks the binding energy shift from the electrostatic potential. It is noteworthy to mention that in addition to the Fe heteroatom, the TFSI^−^ anion also exhibits binding energy shifts that are observable at an intermediate potential (Supplementary Fig. 14). This further supports that the edge of the electrode/monolayer/electrolyte interface is largely pinned to the electrostatic potential of the electrolyte (*φ*_Electrolyte_).

We now discuss the present work in the context of previous UHV-EC-based XPS studies that have reported electrochemically-induced binding energy shifts. These previous studies typically focus on bare metal electrodes restricted to the double-layer charging region^2,26,38,60,61^, whereas in our case, we utilise a reversible redox-active surface-bound molecular probe (Fc SAM). This difference has distinct advantages. The Fc SAM molecular probe offers a controlled, systematic approach since the number of Fc/Fc^+^ redox centres is well-defined throughout the experiments (4.5 × 10^−10^ mol cm^−2^) thus, allows the electrochemical response (CV, charge transferred, and polarisation potential) to be directly correlated and verified with XPS/UPS regarding (1) the degree of Fc^+^–X^−^ ion-pairing (anion coverage), (2) change of Fc/Fc^+^ redox centre (Fe oxidation state), and (3) binding energy shifts (Fe, anion, and valence spectra). This corroborative approach allows for more complete conclusions. Additionally, the construct of the Fc SAM molecular probe is beneficial because the alkyl chain physically and electronically separates the Fc termini from the electrode surface. This circumvents the challenges of existing studies where the influence of the high electric fields on species at the electrode surface can lead to ambiguities in the interpretation of the observed binding energy shifts^61,62^.

We add that the approach described here opens the door to other compelling opportunities including the modulation of the SAM structure, organic ions with strategically positioned heteroatoms^9,63^, use of ionic liquids as a coating or electrolyte^55^, or semiconducting electrodes^64,65^, which will alter the energetics of the electrode/monolayer/electrolyte interface. Our work shows a framework that can be extended to interrogate electrolyte effects and interfacial energetics in redox-active polymers^66^ and in applications relating to functionalised electrodes for pseudocapacitive energy storage^67^.

Using EC-XPS/UPS as a “beyond cyclic voltammetry” approach enables a unique spectroelectrochemical description of the anion-related electronic and structural properties of the electrode/monolayer/electrolyte interface which are not readily assessable with other methods. Upon anodic polarisation, we can spectroscopically discriminate differences in Fc^+^–X^−^ ion-pair composition, monolayer thickness and degree of conversion to Fc^+^ arising from Fc^+^–X^−^ interactions and steric constraints, which manifests in the apparent formal potential and integral charge transferred as observed by CV. Upon subsequent cathodic polarisation corresponding to the conversion back to Fc, our observations are reversible. We have shown that the Fc SAM molecular probe construct enables a useful corroborative approach where the electrochemical response can be readily correlated to the XPS/UPS results allowing for the unobscured observation of the electrostatic potential at the electrode/monolayer/electrolyte interface. This work is part of our ongoing efforts to exploit UHV-EC methods to obtain a richer description of the electrode/electrolyte interface.

## Methods

### Monolayer and substrate preparation

The cylindrical hat-shaped Au(111) substrates (99.999%, MaTecK) with a diameter of 6 mm were cleaned by soaking in piranha solution (concentrated H_2_SO_4_ (Kanto) and 30% H_2_O_2_ (Wako) in a 3:1 volume ratio) at room temperature for at least 3 h. Caution should be exercised because piranha solution is highly corrosive and vigorously reacts with organics meaning that a fume hood and uncovered vessel should be used. After soaking in piranha solution, the Au substrate was rinsed with copious amounts of Milli-Q water (18.2 MΩ, Millipore), dried under N_2_ gas flow and then flame annealed under a butane flame. The clean Au substrates were immersed for at least 12 h in ethanol (Wako) solutions containing 0.1 mM 11-ferrocenyl-1-undecanethiol (Dojindo Laboratories) followed by rinsing with ethanol and drying under a stream of N_2_ gas. For the mixed SAM/dilution experiments, an as-prepared SAM from 1 mM 1-decanethiol (Aldrich) in ethanol was subsequently submerged in 0.1 mM 11-ferrocenyl-1-undecanethiol (in ethanol) for at least 30 mins followed by rinsing with ethanol and drying under a stream of N_2_ gas. The Au(111) roughness factor was found to be good agreement with the literature which was determined to be ~1.3, based on the charge for the reduction of Au oxide (444 μC cm^−2^)^68^.

### Photoelectron spectroscopy combined with an electrochemical cell

Our setup of X-ray and ultraviolet photoelectron spectroscopy combined with and electrochemical cell (EC-XPS/UPS) is similar in working principle to existing UHV-EC reports that involve electrochemistry in a solution environment followed by transfer to a vacuum environment for analysis (Fig. 2a and Supplementary Fig. 1)^2,24,25,69^. The XPS/UPS analysis chamber is connected via gate valve to an EC chamber allowing for sample transfer and electrochemical measurements without exposure to air. The EC chamber contains a retractable PTFE (polytetrafluorethylene) cell where electrochemical measurements are performed under Ar atmosphere (99.999%, Tomoe Shokai). The cell utilises a hanging meniscus setup connected via PFA (perfluoroalkoxy alkane) tubing to a syringe containing the electrolyte. The typical EC-XPS/UPS experiment involves (1) Transfer of Fc SAM on Au sample to the electrochemical (EC) chamber under vacuum (2) Venting the EC chamber with Ar (3) Insertion of cell with electrolyte allowing for electrode immersion and electrochemical characterisations (4) Removal of the immersed electrode from the electrolyte solution under potential control (5) EC chamber evacuation and subsequent transfer to the XPS/UPS analysis chamber. The EC chamber evacuation and transfer occurs in ~10 min. The XPS/UPS (Theta Probe, Thermo Fisher Scientific) consists of a monochromatic Al *Kα* X-ray source (*hv* = 1486.6 eV) with a detection angle of 53° relative to the normal. The base pressure of the XPS/UPS analysis chamber was ~10^−7^ Pa. XPS was performed at room temperature (298 K) using a pass energy setting of 100 eV. The binding energies are referenced to the Au 4f_7/2_ peak at 84.0 eV. Spectral fitting was done using Advantage 5.52 (Thermo Fisher) software using a Shirley background subtraction with Lorentzian and Gaussian ratio of 70:30^55^. Scofield sensitivity factors^70^ were used for quantification. The UV source uses He (I) excitation (21.2 eV). UPS was performed with pass energy of 2 eV with the spectra referenced to the Fermi edge of Au at 0 eV. A bias of −10 V was applied to resolve the secondary electron cutoff. The CV and chronoamperometry electrochemical measurements (HZ-7000 potentiostat, Hokuto Denko) were performed at room temperature (25 °C). The electrolytes consisted of 0.1 M NaTFSI (sodium bis(trifluoromethanesulfonyl)imide, >98%, TCI Chemicals), NaPF_6_ (99.7%, Wako) or NaClO_4_ (99.99% NaClO_4_·H_2_O, Merck) that were prepared using DI water. The electrolytes were bubbled with Ar for at least 25 min prior to use. The counter electrode consists of a coiled Pt wire and the reference electrode is an AuO_*x*_ wire with the data converted to Ag/AgCl (sat. KCl) for comparison.

## Supplementary information

Supplementary information

## Data Availability

The data that support the findings of this study are available from the corresponding authors upon reasonable request.
