# Supplementary material for: Probing consequences of anion-dictated electrochemistry on the electrode/monolayer/electrolyte interfacial properties
Source: Nat Commun. 2020 Aug 21;11:4194. doi: 10.1038/s41467-020-18030-6 (PMC7442636; doi:10.1038/s41467-020-18030-6)
Supplement: Supplementary file 1 — Supplementary information [file 41467_2020_18030_MOESM1_ESM.pdf]

Supplementary Information

Probing consequences of anion-dictated electrochemistry on the electrode/monolayer/electrolyte interfacial properties

Wong *et al.*

Supplementary Note 1: EC-XPS/UPS relative to ambient pressure XPS (AP-XPS):

It is worthwhile to discuss the motivation and advantages of UHV-EC approach involving electrode immersion and transfer (referred here as EC-XPS/UPS), in the context of alternative techniques, namely *in situ* ambient-pressure XPS (AP-XPS).[1-6]

EC-XPS/UPS enables the valence structure of the electrode/monolayer/electrolyte interface to be clearly resolved. This is due to the photon energy used in UPS (*He(I)=21.2 eV*) which exclusively excites valence electrons. In our work, we can resolve the electrochemically-induced changes in the valence spectra (Fc HOMO, SAM and anion). To the extent of our knowledge, we are not aware of existing reports on in situ UPS. This is due to the higher photon energies (2 to 10 keV) and higher inelastic mean free path (λ) needed for AP-XPS to penetrate the electrolyte layer.[1] As a result of this, the higher photon energy invariably includes core-level photoemission and inhibits the ability to fully resolve the valence structure.

Another aspect is that EC-XPS/UPS can offer improved signal-to-noise for the interfacial features of interest because electrode immersion and transfer "unzips" and eliminates excess bulk electrolyte.[7] On the other hand, AP-XPS which operate at higher pressures (torr range)[1,5] has complexities associated with signal attenuation from elastic and inelastic scattering due to the presence of an electrolyte layer and gaseous species. In addition to signal attenuation, the spectra of interest will contain additional features arising from the electrolyte layer and gaseous species in addition to their interfaces, which need to be accounted for during data interpretation. AP-XPS typically requires the careful optimisation of creating stable electrolyte thin films, X-ray energy, chamber pressures, and detection conditions.[1]

Lastly, we note that the EC-XPS/UPS approach used here and *in situ* AP-XPS can be effective complementary techniques because of their respective trade-offs such as the ability to probe the valence structure and good signal-to-noise with EC-XPS/UPS, and improved temporal resolution with in situ AP-XPS.

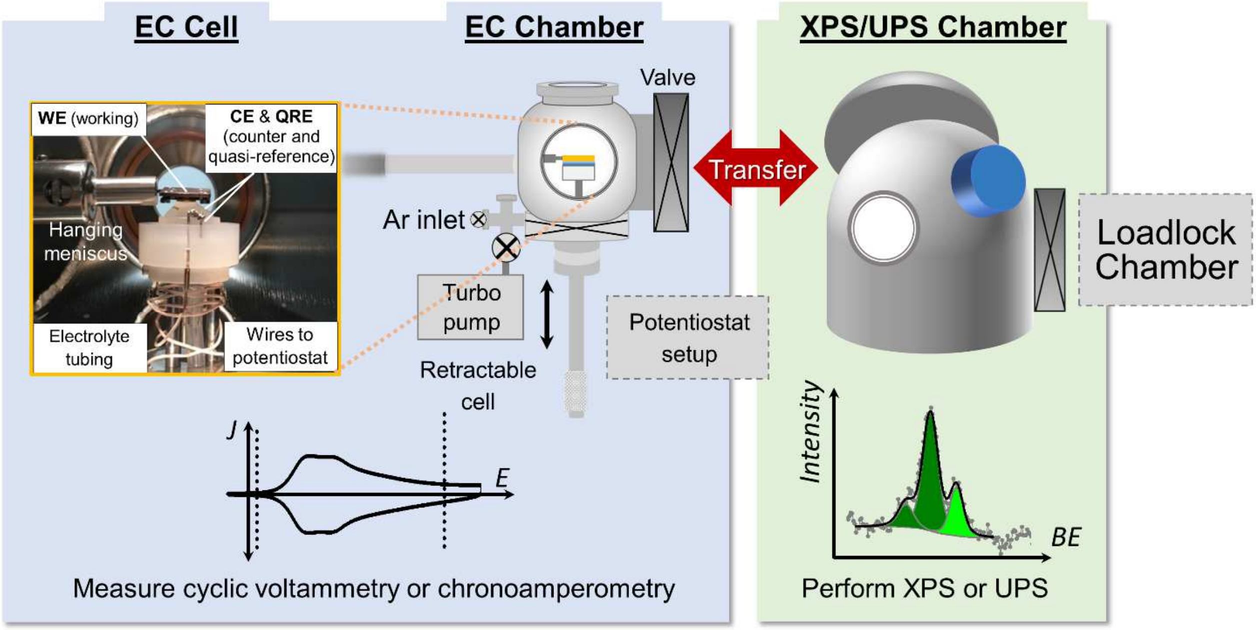

Supplementary Figure 1. Schematic of experimental setup comprised of X-ray and ultraviolet photoelectron spectroscopy combined with an electrochemical cell (EC-XPS/UPS). Electrochemical measurements are performed inside a dedicated "EC chamber" using a hanging meniscus configuration under Ar atmosphere followed cell retraction under potential control, chamber evacuation and then sample transfer. The XPS/UPS analysis chamber connected to the EC chamber *via* gate valve and enables sample transfer back and forth between the analysis chamber and EC chamber.

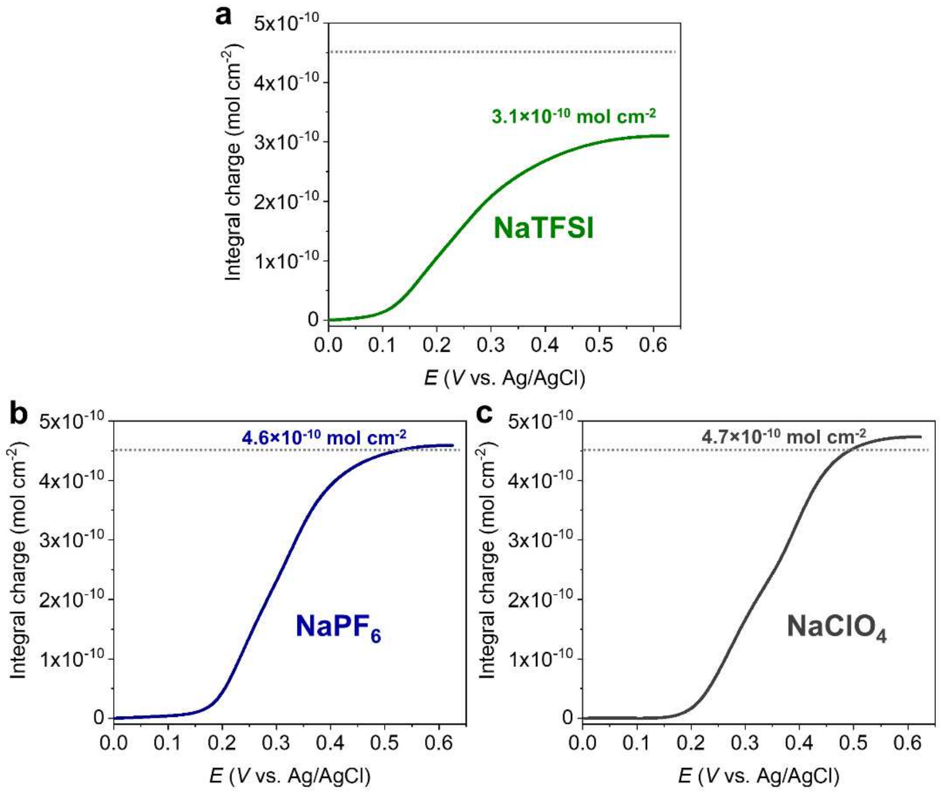

Supplementary Figure 2. Integral charge transferred ($\Gamma_{Fc\rightarrow Fc^{+}}$) as a function of electrode potential analysed using linear background subtraction. The data correspond to the cyclic voltammograms in Figure 2b in the main text. Performed in 0.1 M (a) NaTFSI, (b) $NaPF_6$ and (c) $NaClO_4$. The horizontal dotted line denotes the theoretical Fc SAM coverage of 4.5×10^{-10} mol cm^{-2}.[8]

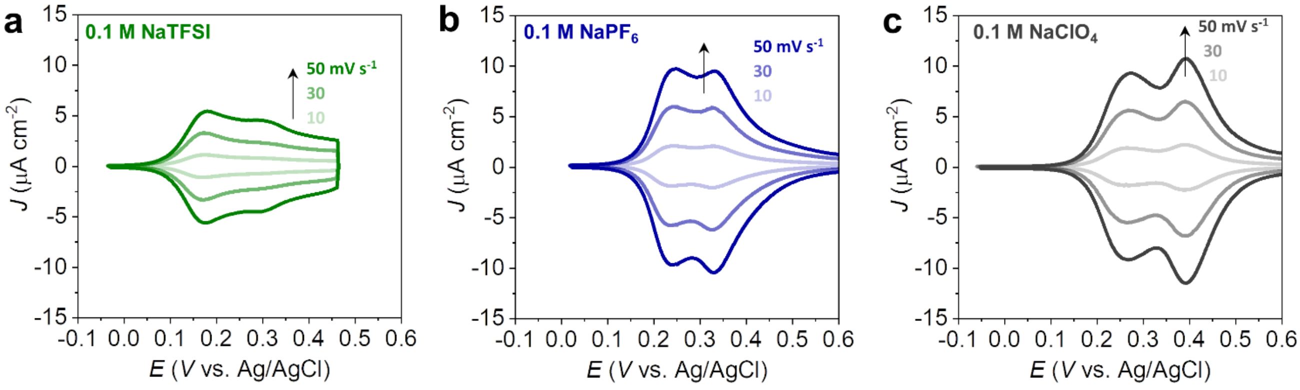

Supplementary Figure 3. Cyclic voltammograms corresponding to 0.1 M (a) NaTFSI, (b) $NaPF_6$ and (c) $NaClO_4$ at the scan rates of 10, 30 and 50 mV s^{-1}.

Supplementary Note 2: Cyclic voltammograms (CVs) and non-ideal behaviour

All of the CVs in Figure 2b of the main text corresponds to a reversible surface-bound redox reaction. This is evidenced by the near negligible anodic to cathodic peak separations (< 5 mV) and peak currents that scale linearly with scan rate. We note that purification of the Fc SAM *via* silica-gel column chromatography resulted in the same non-ideal behaviour with CVs that were virtually identical indicating that impurities are unlikely the origin of the non-ideal behaviour.

The ideal surface-bound Nernstian reaction exhibits a CV that contains a single peak (FWHM of 90.6/*n* mV, *n* is the number of electrons) and negligible peak-to-peak separation. However, Fc SAM at higher coverages commonly exhibit deviations from ideal behaviour with asymmetric/multiple peaks and peak broadening.[8-10] In terms of the origin of the non-ideal behaviour, there have been several proposed origins including:

(1) Heterogeneity at the local level with different structured/packed domains[11] or so-called isolated and clustered Fc.[9] Rudnev *et al*[12] performed a CV and scanning probe microscopy investigation of low-index single crystal and polycrystalline Au, to show that the CV response is related to differences Fc SAM ordering (local disordering and ordered domains), and this further depends on the crystallographic surface structure. The Fc SAM coverage can be diluted with non-electroactive *n*-alkanethiols to yield CVs with characteristics that are more ideal.

(2) Along a similar thread is the nature of the intermolecular interactions experienced by the Fc termini. For surface-bound redox-active monolayers, the CVs can be fitted to phenomenological models based on the Langmuir or Frumkin isotherms to provide insights into the nature of the intermolecular interactions.[11,13] A FWHM of 90.6/n mV corresponds to the absence of lateral interactions (between oxidised and reduced forms of Fc, i.e. O-O, R-R and O-R) whereas an FWHM that is greater or smaller than 90.6/n mV corresponds to attractive and repulsive interactions, respectively.

(3) Buried Fc termini[14] and double-layer effects.[15,16] As experimentally shown by Rowe and Creager[17] and modelled by Smith and White,[18] positional differences between the plane of electron transfer (PET) and the closest approach of ions can lead to so-called double-layer effects causing the multiplicity and broadening of the CV peaks.[16] For instance, it has been suggested that due to the mismatch between the bulkier Fc termini and the alkyl chain, the resulting strain can cause the presence of buried Fc[14] which can result in multiple PET (plane of electron transfer) and thus leads to non-ideal behaviour.

We note that other methods to obtain more ideal CVs include the inclusion of polar functional groups[8] or utilization of a rigid spacer.[19]

Supplementary Table 1. XPS-determined stoichiometry for pristine Fc SAM and after polarization at $E_{(anodic)}$ and $E_{(cathodic)}$ showing the formation of 1:1 Fc^{+}–X^{-} ion-pairs upon oxidation to Fc^{+}. The similarity of the Fe 2p area ratios) indicates the reversibility of the EC-XPS/UPS method following electrochemistry and sample transfer. The tabulated data corresponds to the spectra in Figures 3 and S10.

		Fe:N:F (atomic ratio)	Fe 2p (area ratio)			Fe:P:F (atomic ratio)	Fe 2p (area ratio)
**$TFSI^{-}$**	Pristine	1:0:0	1.0	**PF_6^{-}**	Pristine	1:0:0	1.0
	$E_{(anodic)}$	1.2:1.0:6.0	0.97		$E_{(anodic)}$	0.9:1.0:6.3	0.97
	$E_{(cathodic)}$	1:0:0	0.95		$E_{(cathodic)}$	1:0:0	0.94

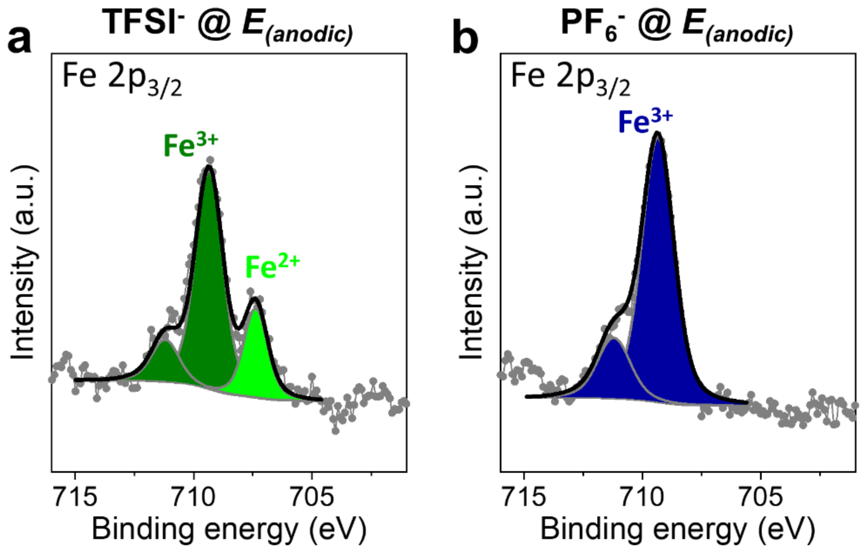

Supplementary Figure 4. EC-XPS Fe $2p_{3/2}$ spectra following $E_{(anodic)}$ showing the deconvoluted Fe $2p_{3/2}$ contributions from Fe^{3+} and Fe^{2+} for (a) $TFSI^{-}$ and (b) PF_6^{-}. In the case of $TFSI^{-}$, the area ratio indicates that ~77% of the Fc has oxidised to Fc^{+}.

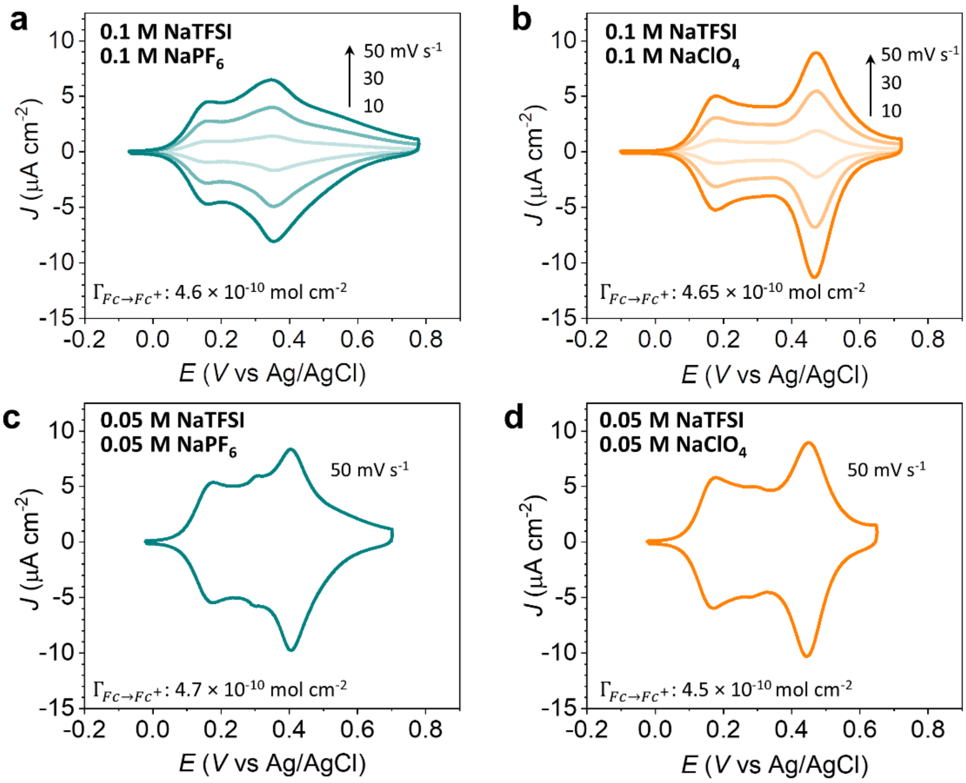

Supplementary Figure 5. Cyclic voltammograms in the presence of competitive (mixed anion) ion pairing, performed in (a) 0.1 M NaTFSI and 0.1 M $NaPF_6$, (b) 0.1 M NaTFSI and 0.1 M $NaClO_4$. To observe if there are concentration effects, additional CVs were performed in (c) 0.05 M NaTFSI and 0.05 M $NaPF_6$ (d) 0.05 M NaTFSI and 0.05 M $NaClO_4$. We note that we can observe a Nernstian shift (~59 mV dependence on concentration) with respect to the first set of CV peaks while the other peaks do not exhibit Nernstian shifts. The origin of this behaviour is the focus of a future investigation. Nonetheless, the degree of conversion to Fc^+ as indicated by $\Gamma_{Fc\rightarrow Fc^+}$ in each figure equates to ~4.5-4.7×10^{-10} mol cm^{-2} (theoretical Fc coverage is 4.5×10^{-10} mol cm^{-2}), which suggests that due to steric constraints of $TFSI^-$ anions, full conversion to Fc^+ (seen in Figure 2b of main text) is restricted. On the other hand, in the presence of smaller anions (PF_6, ClO_4^-) the full conversion to Fc^+ can proceed.

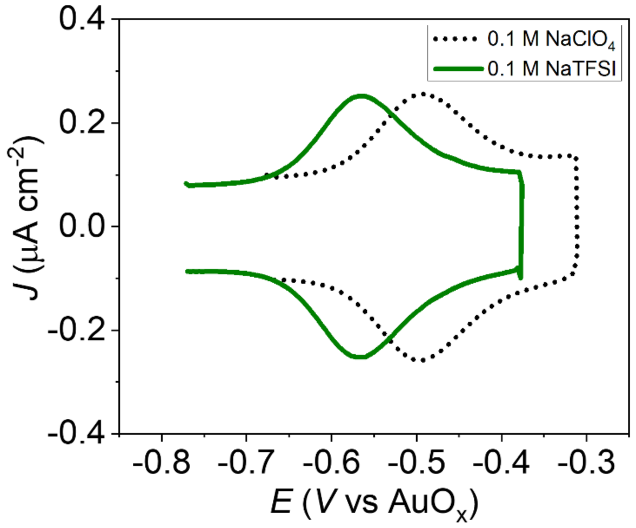

Supplementary Figure 6. Cyclic voltammograms showing Fc SAM diluted with non-electroactive alkanethiols (1-decanethiol) performed in (a) 0.1 M NaTFSI and (b) $NaClO_4$ at a scan rate of 50 mV s^{-1}.The comparable charge transferred (integral area) indicates that $\Gamma_{Fc\rightarrow Fc^{+}}$ does not exhibit anion dependencies at reduced Fc SAM coverages.

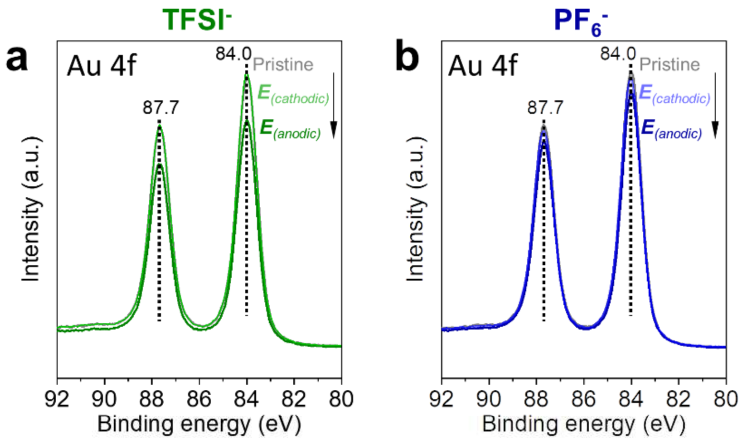

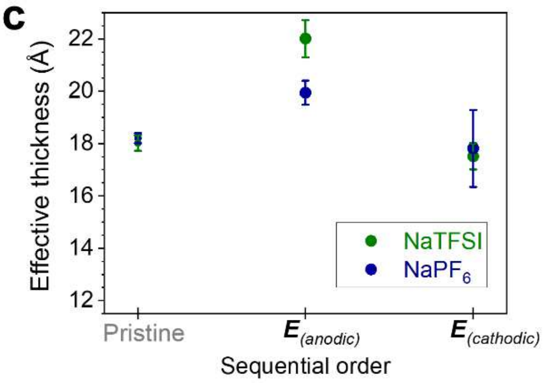

	Pristine (Å)	$E_{(anodic)}$ (Å)	$E_{(cathodic)}$ (Å)
**$TFSI^-$**	18.0 ± 0.3	22.0 ± 0.7	17.5 ± 0.5
**PF_6^-**	18.2 ± 0.2	19.9 ± 0.5	17.8 ± 1.5

Supplementary Figure 7. EC-XPS of Fc SAM following $E_{(anodic)}$ and $E_{(cathodic)}$ showing the Au 4f spectra and corresponding change in effective thickness. Performed in 0.1 M (a) NaTFSI and (b) $NaPF_6$. (c) Change in effective thickness following polarization at $E_{(anodic)}$ and $E_{(cathodic)}$ based on the attenuation differences in the Au 4f spectra (See supplementary note 3 on the method used to determine effective thickness and additional discussion). Error bars indicate SD based on at least 3 independent measurements. The effective thickness of ~18 Å for the pristine Fc SAMs is in line with existing reports utilising other methods.[20,21] The effective thicknesses increases upon $E_{(anodic)}$ for both anions with $TFSI^-$ at 22.0 ± 0.7 Å and PF_6^- at 19.9 ± 0.5 Å, respectively. Although $TFSI^-$ is not spherical in nature, the effective ionic radii can be approximated from the van der Waals volume of the anions, where the effective ionic radii then equates to 0.33 and 0.25 nm for $TFSI^-$ and PF_6^- respectively.[22] Assessing these values, if we assume that the anion is positioned on top of the Fc SAM, the 0.16 nm difference in effective ionic diameters between $TFSI^-$ and PF_6^- is within the error bar ranges of our effective thickness following $E_{(anodic)}$. However, we note that in reality, the precise position of the anion is not known. We note that the increase in effective thickness should also include a contribution from a change in Fc SAM molecular orientation (i.e. reorientation of Fc^+ termini or alkyl chain)[23,24] that have been reported to occur upon oxidation to Fc^+. In our case, and in particular with $TFSI^-$, the change in effective thickness between $E_{(anodic)}$ and $E_{(cathodic)}$ (~4 Å) is larger than the magnitude of the orientation changes reported ranging from 0.09 to 1.9 Å,[23,25] indicating that the anion should be contributing to the observed increase in effective thickness.

Supplementary Note 3: Effective thickness (d_{SAM}) from Au 4f attenuation:

The effective thickness (d_{SAM}) can be determined using the general equation of the attenuation of photoelectrons caused by inelastic scattering in the presence of an adlayer over a substrate as shown in Supplementary equation 1.

$$I_{SAM} = I_{Au} \exp\left(-\frac{d_{SAM}}{\lambda_{SAM}}\right) \qquad \text{(Supplementary equation 1)}$$

where I_{SAM} and I_{Au} correspond to the intensity of the SAM covered and bare Au substrate, respectively and λ_{SAM} is the attenuation length. The attenuation length has been determined experimentally as shown by Bain and Whitesides[26] by systematically measuring *n*-alkanethiol ($C_nH_{2n+1}SH$) SAM with increasing thicknesses. Additionally, the attenuation length can be estimated as shown by Kondo et al using equations based on TPP-2M methods[27] or by using the relation proposed by Seah and Dench for the attenuation length of a generic organic compound.[28] A comparison of the attenuation lengths from Bain and Whitesides was found to yield similar values to TPP-2M, which yielded thicknesses that are in good agreement with existing reports utilizing other methods. Therefore, we have used the attenuation lengths from Bain and Whitesides to determine the effective thickness following $E_{(anodic)}$ and $E_{(cathodic)}$ presented in Supplementary Figure 7.

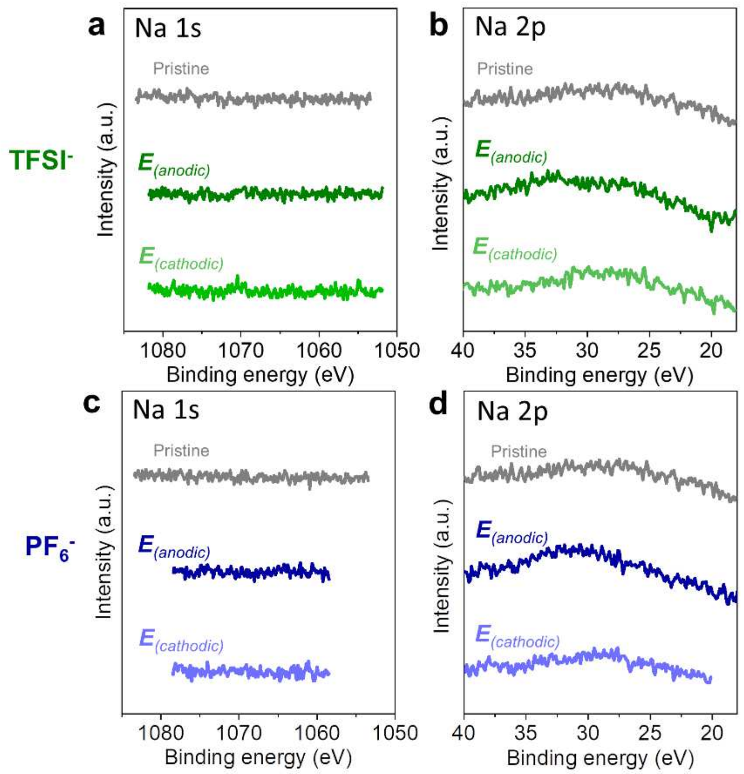

Supplementary Figure 8. EC-XPS of pristine Fc SAM and following $E_{(anodic)}$ and $E_{(cathodic)}$ corresponding to 0.1 M NaTFSI (a) Na 1s (b) Na 2p, and 0.1 M $NaPF_6$ (c) Na 1s, and (d) Na 2p spectra.

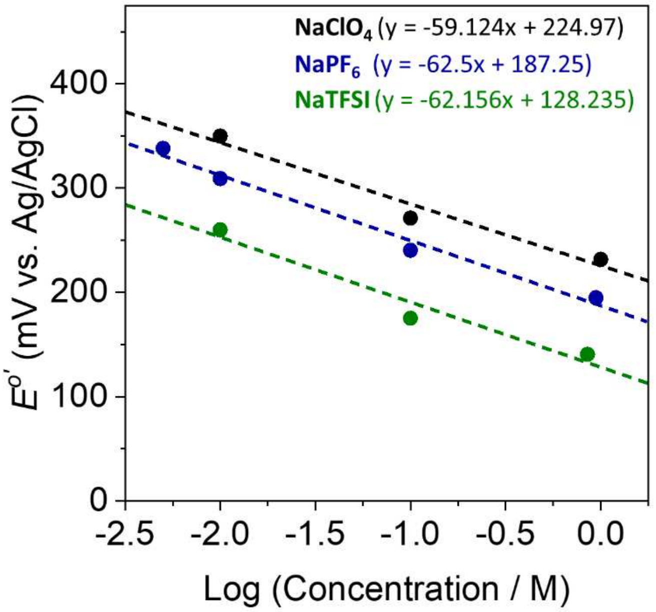

Supplementary Figure 9. Apparent formation potential ($E^{o'}$) as a function of anion concentration in NaTFSI, $NaPF_6$ and $NaClO_4$. The apparent formal potential is defined as the average of the first set of anodic and cathodic peak potentials as obtained from cyclic voltammetry.

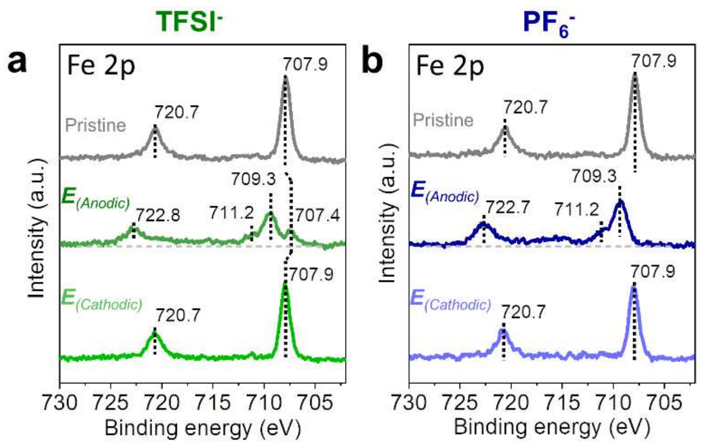

Supplementary Figure 10. EC-XPS of the pristine Fc SAM and following polarization at $E_{(anodic)}$ and $E_{(cathodic)}$ showing the full range of the Fe 2p spectra performed in 0.1 M (a) NaTFSI and (b) $NaPF_6$.

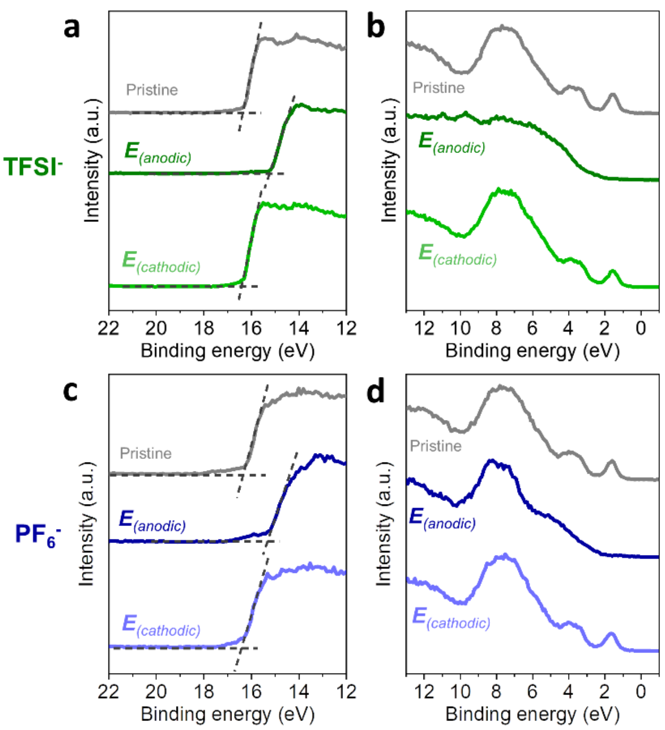

Supplementary Figure 11. EC-UPS spectra of pristine Fc SAMs and following $E_{(anodic)}$ and $E_{(cathodic)}$ showing the spectral regions near the secondary electron cutoff and Fermi level (0 eV). Electrolyte corresponds to (a-b) 0.1 M NaTFSI and (c-d) $NaPF_6$, respectively. Following $E_{(anodic)}$, there is a distinct difference between $TFSI^-$ and PF_6^- in the extended valence structure. This indicates that the uniqueness of the Fc^+–$TFSI^-$ ion-pair structure can promote the screening of the SAM/Au spectra features. For example, in a situation where bulky $TFSI^-$ anions are positioned on top of the Fc^+, the SAM/Au spectral features are expected be screened. In the case of PF_6^-, the SAM/Au features remain prominent after $E_{(anodic)}$ (Supplementary Figure 11) despite having a higher anion coverage. This suggests that in comparison to $TFSI^-$, the position of the PF_6^- anions within the Fc^+–PF_6^- ion-pair structure, has less of an influence on SAM/Au photoemission.

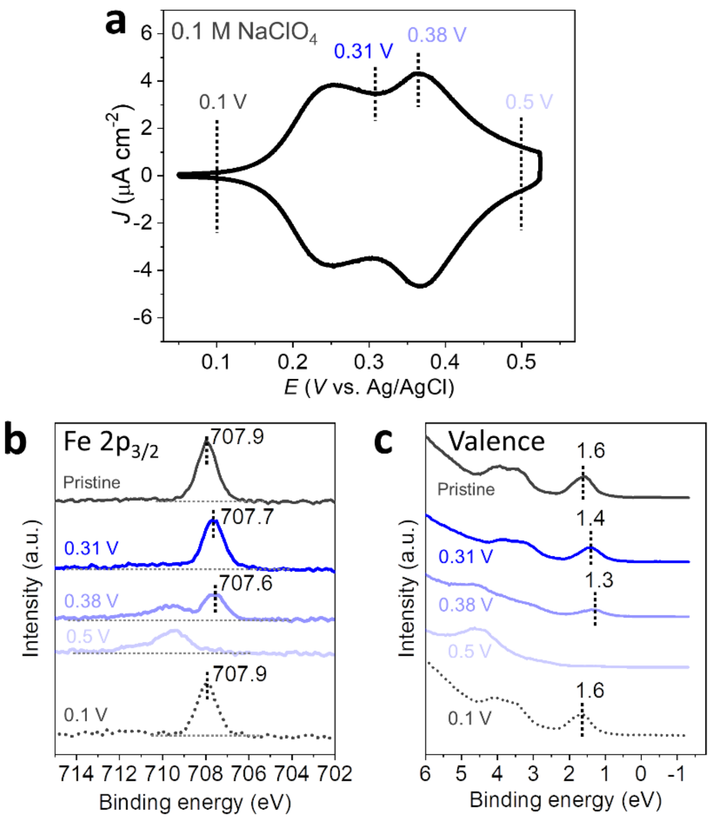

Supplementary Figure 12. EC-XPS/UPS measurements of Fc SAM at increasing potentials for 0.1 M $NaClO_4$. (a) CV performed at 20 mV s^{-1}. The potentials at which the electrode was polarised is indicated by the vertical dotted lines. (b) XPS Fe $2p_{3/2}$ and (c) UPS valence spectra regions (Fermi level is 0 eV).

Supplementary Note 4: Theoretical description of interfacial potential distribution

In regards to the interfacial potential distribution, the overwhelming majority of the potential drop occurs within the monolayer. This is due to the (1) presence of ion-pairing which neutralises/screens the surface charge,[29] and (2) large difference in the relative permittivity of the SAM (ε = 3) compared to the electrolyte (ε = 78.5).[30] Theoretical descriptions of a surface-bound redox-active monolayer can provide insights into the parameters that affect the interfacial potential distribution.[18,30] Using a similar method to Smith and White[18,31] for a redox-active monolayer (Supplementary Figure 13a), we can show how ion-pairing affects the electrostatic potentials ($\varphi_{Electrode}$, φ_{PET}, φ_F) as a function of electrode potential (E vs E_{pzc})(Supplementary Figure 13b-c). In the case of ion-pairing, there is an additional parameter (X) which affects the charge density of σ_{PET} as shown in the following relation (Supplementary equation 2):[31]

$$\sigma_{PET} = (1 - X)(F\Gamma_T z f) \qquad \text{(Supplementary equation 2)}$$

where X is between 0 to 1 and represents how effectively the surface charge at the PET is compensated. Other parameters include F (Faraday constant), Γ_T is the coverage (in mol m^{-2}), z is valence of the oxidised state, and f is the fraction of oxidised species ($f = \Gamma_{oxidised} / \Gamma_T$). The parameters used for Supplementary Figure 13 are ε_1 = 3, ε_2 = 12, ε_3 = 78.5, d_1 = 2.2 nm (height of chain length of 11 hydrocarbons plus radius of Fc),[8,32] d_2 = 0.1 nm (typical size of a water molecule), z = 1, electrolyte concentration = 0.1 M, $\varphi_{Electrolyte}$= 0 V, E^o = 0.2 V, E_{pzc} = 0 V, Γ_T = 0.5×10^{-10} mol cm^{-2}, and X = 0.02 in Supplementary Figure 13c.

We can observe that in the presence of ion-pairing (Supplementary Figure 13c), the potential drop is predominantly within the monolayer and there is a negligible influence from φ_{PET}, φ_F irrespective of electrode potential. We note that electrolyte concentration does not significantly change the potential distribution in the concentrations of interest (0.1 to 1 M). In terms of the Fc SAM overage, increasing the coverage results in the same tendencies. A key parameter is X as this determines the effectiveness of ion-pairing and charge neutralisation while φ_{PET} becomes appreciable at X > 0.15.

It is noteworthy to mention the importance of d_2. In the presence of buried Fc, the value of d_2 will be affected which in turn will influence the interfacial potential distribution. Calvente *et al.*[16] has theoretically shown the interfacial potential distribution in the presence of multiple redox-planes. Nonetheless, the position of the Fe heteroatom within the monolayer will affect the binding energy shifts and can be related to the deviations from the expected 1 eV/V relationship.

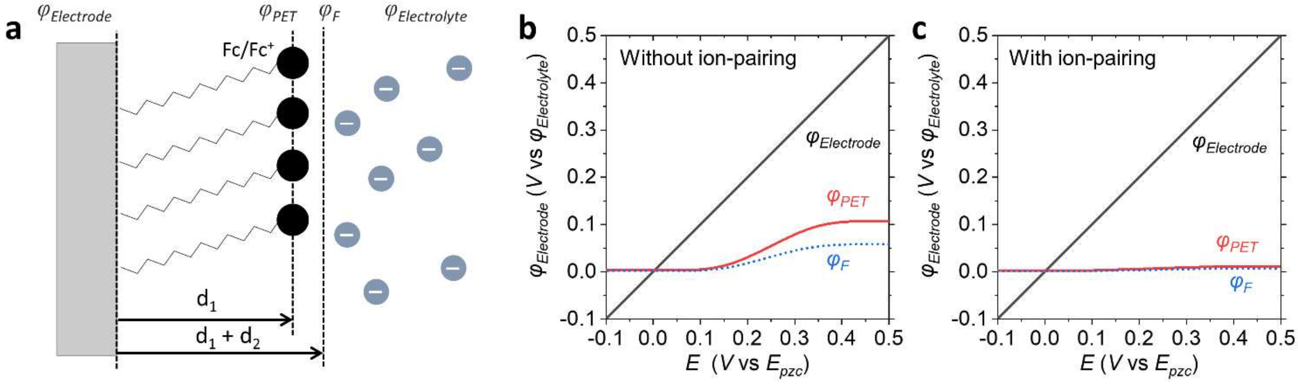

Supplementary Figure 13. Calculations of the interfacial potential distribution using a similar method to Smith and White[18,31]. (a) Schematic of the interface showing the parameters in the calculation. (b) Dependence of $\varphi_{Electrode}$, φ_{PET}, φ_F on electrode potential *[E]* without ion-pairing, and (c) with ion-pairing. PET denotes plane of electron transfer.

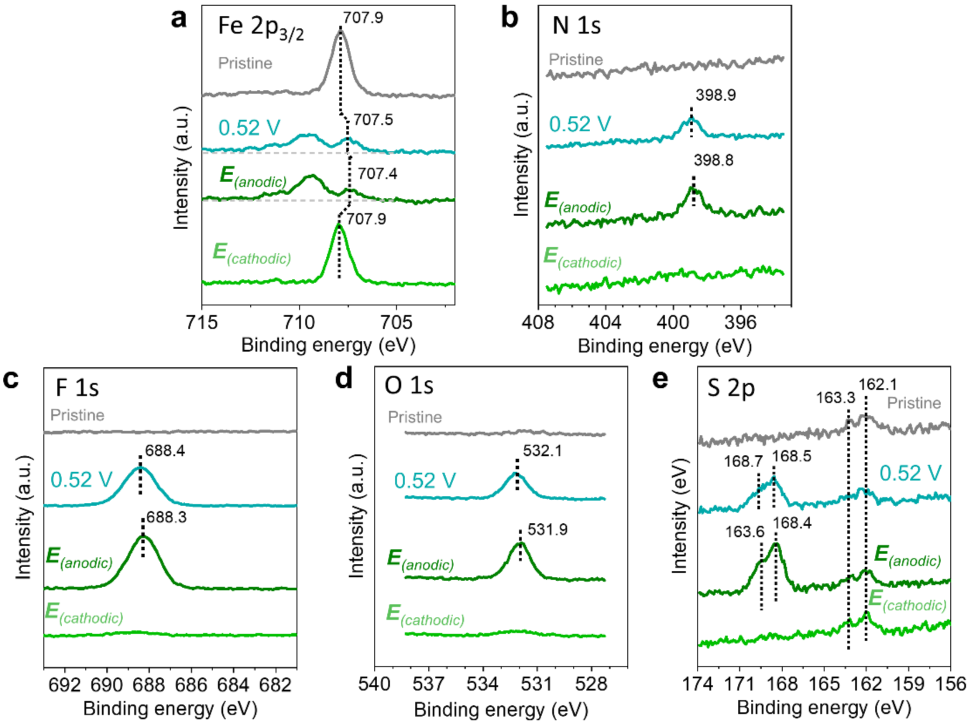

Supplementary Figure 14. EC-XPS of pristine Fc SAM and following polarisation at 0.52 V, E(anodic) and E(cathodic) in 0.1 M NaTFSI. (a) Fe 2p (b) N 1s (c) F 1s (d) O 1s, and (e) S 2p spectra. The pristine, $E_{(anodic)}$ and $E_{(cathodic)}$ spectra correspond to the data in the main text (Figures 3, 5 and 7). $E_{(anodic)}$ and $E_{(cathodic)}$ correspond to 0.625 and 0 V vs Ag/AgCl, respectively. Note that the different signal-to-noise in (b) is due to different data acquisition settings.

Supplementary References

1 Favaro, M. *et al.* Interface science using ambient pressure hard X-ray photoelectron spectroscopy. *Surfaces* **2**, 78-99 (2019).

2 Salmeron, M. & Schlögl, R. Ambient pressure photoelectron spectroscopy: A new tool for surface science and nanotechnology. *Surf. Sci. Rep.* **63**, 169-199 (2008).

3 Ketteler, G. *et al.* The nature of water nucleation sites on TiO_2 (110) surfaces revealed by ambient pressure X-ray photoelectron spectroscopy. *J. Phys. Chem. C* **111**, 8278-8282 (2007).

4 Crumlin, E. J. *et al.* X-ray spectroscopy of energy materials under in situ/operando conditions. *J. Electron Spectrosc. Relat. Phenom.* **200**, 264-273 (2015).

5 Stoerzinger, K. A., Hong, W. T., Crumlin, E. J., Bluhm, H. & Shao-Horn, Y. Insights into electrochemical reactions from ambient pressure photoelectron spectroscopy. *Acc. Chem. Res.* **48**, 2976-2983 (2015).

6 Casalongue, H. S. *et al.* Direct observation of the oxygenated species during oxygen reduction on a platinum fuel cell cathode. *Nat. Commun.* **4**, 1-6 (2013).

7 Bockris, J. O. M. & Khan, S. U. *Surface electrochemistry: a molecular level approach*. (Plenum Press, 1993).

8 Chidsey, C. E., Bertozzi, C. R., Putvinski, T. & Mujsce, A. Coadsorption of ferrocene-terminated and unsubstituted alkanethiols on gold: electroactive self-assembled monolayers. *J. Am. Chem. Soc.* **112**, 4301-4306 (1990).

9 Lee, L. Y. S., Sutherland, T. C., Rucareanu, S. & Lennox, R. B. Ferrocenylalkylthiolates as a probe of heterogeneity in binary self-assembled monolayers on gold. *Langmuir* **22**, 4438-4444 (2006).

10 Yokota, Y. *et al.* Electronic-state changes of ferrocene-terminated self-assembled monolayers induced by molecularly thin ionic liquid layers: a combined atomic force microscopy, x-ray photoelectron spectroscopy, and ultraviolet photoelectron spectroscopy study. *J. Phys. Chem. C* **119**, 18467-18480 (2015).

11 Tian, H., Dai, Y., Shao, H. & Yu, H.-Z. Modulated Intermolecular Interactions in Ferrocenylalkanethiolate Self-Assembled Monolayers on Gold. *J. Phys. Chem. C* **117**, 1006-1012 (2013).

12 Rudnev, A. V., Yoshida, K. & Wandlowski, T. Electrochemical characterization of self-assembled ferrocene-terminated alkanethiol monolayers on low-index gold single crystal electrodes. *Electrochim. Acta* **87**, 770-778 (2013).

13 Vogel, Y. B. *et al.* Reproducible flaws unveil electrostatic aspects of semiconductor electrochemistry. *Nat. Comm.* **8**, 2066 (2017).

14 Nerngchamnong, N. *et al.* Nonideal electrochemical behavior of ferrocenyl–alkanethiolate SAMs maps the microenvironment of the redox unit. *J. Phys. Chem. C* **119**, 21978-21991 (2015).

15 Creager, S. E. & Rowe, G. K. Solvent and double-layer effects on redox reactions in self-assembled monolayers of ferrocenyl—alkanethiolates on gold. *J. Electroanal. Chem.* **420**, 291-299 (1997).

16 Calvente, J. J., Andreu, R., Molero, M., López-Pérez, G. & Domínguez, M. Influence of spatial redox distribution on the electrochemical behavior of electroactive self-assembled monolayers. *J. Phys. Chem. B* **105**, 9557-9568 (2001).

17 Rowe, G. K. & Creager, S. E. Interfacial solvation and double-layer effects on redox reactions in organized assemblies. *J. Phys. Chem.* **98**, 5500-5507 (1994).

18 Smith, C. P. & White, H. S. Theory of the interfacial potential distribution and reversible voltammetric response of electrodes coated with electroactive molecular films. *Anal. Chem.* **64**, 2398-2405 (1992).

19 Kitagawa, T. *et al.* Ideal redox behavior of the high-density self-assembled monolayer of a molecular tripod on a Au (111) surface with a terminal ferrocene group. *Langmuir* **29**, 4275-4282 (2013).

20 Norman, L. L. & Badia, A. Microcantilevers modified with ferrocene-terminated self-assembled monolayers: effect of molecular structure and electrolyte anion on the redox-induced surface stress. *J. Phys. Chem. C* **115**, 1985-1995 (2010).

21 Watcharinyanon, S., Moons, E. & Johansson, L. S. Mixed self-assembled monolayers of ferrocene-terminated and unsubstituted alkanethiols on gold: surface structure and work function. *J. Phys. Chem. C* **113**, 1972-1979 (2009).

22 Ue, M. Mobility and ionic association of lithium and quaternary ammonium salts in propylene carbonate and γ-butyrolactone. *J. Electrochem. Soc.* **141**, 3336-3342 (1994).

23 Yao, X., Wang, J., Zhou, F., Wang, J. & Tao, N. Quantification of redox-induced thickness changes of 11-ferrocenylundecanethiol self-assembled monolayers by electrochemical surface plasmon resonance. *J. Phys. Chem. B* **108**, 7206-7212 (2004).

24 Ye, S., Sato, Y. & Uosaki, K. Redox-induced orientation change of a self-assembled monolayer of 11-ferrocenyl-1-undecanethiol on a gold electrode studied by in situ FT-IRRAS. *Langmuir* **13**, 3157-3161 (1997).

25 Feng, Y., Dionne, E. R., Toader, V., Beaudoin, G. & Badia, A. Odd–even effects in electroactive self-assembled monolayers investigated by electrochemical surface plasmon resonance and impedance spectroscopy. *J. Phys. Chem. C* **121**, 24626-24640 (2017).

26 Bain, C. D. & Whitesides, G. M. Attenuation lengths of photoelectrons in hydrocarbon films. *J. Phys. Chem.* **93**, 1670-1673 (1989).

27 Kondo, T., Yanagida, M., Shimazu, K. & Uosaki, K. Determination of thickness of a self-assembled monolayer of dodecanethiol on Au (111) by angle-resolved X-ray photoelectron spectroscopy. *Langmuir* **14**, 5656-5658 (1998).

28 Seah, M. P. & Dench, W. Quantitative electron spectroscopy of surfaces: A standard data base for electron inelastic mean free paths in solids. *Surf. Interface Anal* **1**, 2-11 (1979).

29 Eggers, P. K., Darwish, N., Paddon-Row, M. N. & Gooding, J. J. Surface-bound molecular rulers for probing the electrical double layer. *J. Am. Chem. Soc.* **134**, 7539-7544 (2012).

30 Ohtani, M., Kuwabata, S. & Yoneyama, H. Voltammetric Response Accompanied by Inclusion of Ion Pairs and Triple Ion Formation of Electrodes Coated with an Electroactive Monolayer Film. *Anal. Chem.* **69**, 1045-1053 (1997).

31 Shiota, K. & Osakai, T. The effect of supporting electrolyte on the electron transfer at mixed self-assembled monolayers containing ferrocene moieties. *J. Electroanal. Chem.* **754**, 75-79 (2015).

32 Porter, M. D., Bright, T. B., Allara, D. L. & Chidsey, C. E. Spontaneously organized molecular assemblies. 4. Structural characterization of n-alkyl thiol monolayers on gold by optical ellipsometry, infrared spectroscopy, and electrochemistry. *J. Am. Chem. Soc.* **109**, 3559-3568 (1987).
